# Circular RNA expression profile in transgenic diabetic mouse kidneys

**DOI:** 10.1186/s11658-021-00270-z

**Published:** 2021-06-07

**Authors:** Xuan Xiong, Changchun Liu, Meiren Shen, Qian Yang, Qiang Zhao, Xiaoyan Li, Xiaoshi Zhong, Zhiwei Wang

**Affiliations:** 1 Department of Nephrology, The Affiliated Shunde Hospital of Guangzhou Medical University, Foshan, 528315 China; 2 Department of Nephrology, Guangzhou Red Cross Hospital, Medical School of Jinan University, Guangzhou, 510220 China; 3 Department of Cardiology, The Affiliated Shunde Hospital of Guangzhou Medical University, Foshan, 528315 China; 4 Laboratory Medicine Centre, The Affiliated Shunde Hospital of Guangzhou Medical University, Foshan, 528315 China; 5 Guangzhou Institute of Disease-Oriented Nutritional Research, Guangzhou Red Cross Hospital, Medical School of Jinan University, Guangzhou, 510220 China

**Keywords:** Circular RNA, Diabetic nephropathy, miRNA target, Gene ontology analysis, Pathway analysis

## Abstract

**Background:**

Diabetic nephropathy is one of the most important complications in patients with diabetes. The etiology and pathogenesis of diabetic nephropathy remain unclear. Several studies have indicated that circular RNAs (circRNAs) play crucial regulatory roles in numerous human diseases and normal physiology; however, to date, no study has focused on the comprehensive expression profile of circRNAs in the kidneys of diabetic mice. Therefore, we aimed to identify differentially expressed circRNAs in diabetic mouse kidneys to explore the possible roles of dysregulated circRNAs in diabetic nephropathy development.

**Results:**

Diabetic BKS-Lepr^em2Cd479^/Nju (BKS-DB/Nju) mice and their nondiabetic wild-type littermates of C57BL/KsJ wild-type (WT) mice were used as experimental animals. Among all circRNAs identified by high-throughput RNA sequencing, four circRNAs were upregulated and ten were downregulated in diabetic mouse kidneys compared to those in nondiabetic mouse kidneys. After verification using quantitative reverse transcriptase polymerase chain reaction assays, we found that circR_1084, circR_182, circR_4, circR_50, circR_596, circR_897, and circR_203 were downregulated, whereas circR_627, circR_628, circR_735, and circR_801 were upregulated in the renal tissues of 8- and 16-week-old BKS-DB/Nju mice compared to those in WT mice.

**Conclusion:**

We studied the circRNA expression profile in the kidneys of diabetic mice. Differentially expressed circRNAs may be useful as candidate biomarkers for diabetic nephropathy. Collectively, our results provide a novel theoretical basis for further investigation of the regulatory roles of circRNA in the etiology and pathogenesis of diabetic nephropathy.

**Supplementary Information:**

The online version contains supplementary material available at 10.1186/s11658-021-00270-z.

## Background

Diabetes, a metabolic disease characterized by hyperglycemia [1], is caused by a deficiency in insulin secretion and/or biological impairment. The long-term existence of hyperglycemia in diabetes patients leads to chronic damage and dysfunction of various tissues, particularly the eyes, kidneys, heart, blood vessels, and nerves. According to the Ninth Edition of IDF Diabetes Atlas (https://diabetesatlas.org/en/resources/), released by the International Diabetes Federation in 2019, approximately 463 million people have diabetes and this number is projected to reach 578 million by 2030. Therefore, diabetes not only causes physical and psychological pain in patients but also results in a heavy social and economic burden. Diabetic nephropathy is a common microvascular complication of diabetes [2]. Approximately 40% of patients with type 1 and type 2 diabetes develop nephropathy [3, 4]. Concomitant with the increasing incidence of diabetes, the incidence of diabetic nephropathy continues to increase, and this condition is emerging as the main cause of end-stage kidney disease [2, 5]. Therefore, it is of vital interest to clinicians and researchers to fully understand the etiology and pathogenesis of diabetic nephropathy, as this understanding will be helpful in providing new ideas for the prevention and treatment of diabetic nephropathy.

Circular RNA (circRNA) is a newly identified noncoding RNA [6]. This type of RNA is different from linear RNA, as it possesses a closed circular structure and is not affected by RNA exonucleases [7]. Based on these characteristics, circRNA expression is more stable than that of linear RNA and is difficult to degrade [8]. Several reports have indicated that dysregulated expression of circRNAs plays an important role in the occurrence and development of several human diseases [7, 9–11]. CircRNAs, such as circRNA-HIPK3 [12] and circRNA-cPWWP2A [13], have been identified as regulators of diabetic nephropathy. Additionally, circRNA_15698 has been reported to play a role in regulating diabetic nephropathy by promoting extracellular matrix-related protein synthesis in mesangial cells [14]. However, the roles of circRNAs in the occurrence and development of diabetes, especially diabetic nephropathy, are far from being fully characterized.

In the present study, we aimed to analyze the comprehensive circRNA expression profile in diabetic mouse kidneys, which will be helpful in identifying new markers for the prevention and treatment of diabetic nephropathy.

## Methods

### Animals

Six-week-old diabetic BKS-Lepr^em2Cd479^/Nju (BKS-DB/Nju) mice (n = 23, ~ 34 g) and their nondiabetic wild-type littermates of C57BL/KsJ wild-type (WT) mice (n = 23, ~ 21 g) were obtained from the Model Animal Research Center of Nanjing University (Nanjing, China). All mice were housed under controlled conditions (22 ± 2 °C, 12 h light/dark cycle, and 50 ± 10% humidity). Eight-week-old and 16-week-old BKS-DB/Nju and WT mice were used in all experiments.

At 8 weeks and 16 weeks of age, the body weight, 24-h urine volume, blood glucose, insulin, urine creatinine, urea nitrogen, and 24-h urinary albumin were measured. On the last day of week 8, the mice were euthanized by an intraperitoneal injection of 3% sodium pentobarbital (130 mg/kg of animal body weight), and the renal tissues were collected for periodic acid-Schiff (PAS) staining, high-throughput RNA sequencing, and quantitative reverse transcriptase polymerase chain reaction (qRT-PCR). On the last day of week 16, the mice were euthanized as described above, and the renal tissues were collected for PAS staining and qRT-PCR.

### PAS staining

PAS staining was conducted according to routine protocols. To quantify mesangial expansion, the mesangial matrix and glomerular areas of 10 randomly selected glomeruli from each section were quantified by two pathologists in a double-blinded manner using Image Pro-Plus 6.0 (Media Cybernetics, Rockville, MD, USA). The mesangial matrix comprised the area of the PAS-positive and nuclei-free regions within the mesangium. The glomerular area was defined by tracing along the borders of the capillary loop. The relative mesangial area was evaluated as a fraction of the mesangial matrix area relative to that of the glomeruli.

### RNA isolation and high-throughput RNA sequencing

Renal tissue samples were collected from BKS-DB/Nju (n = 3) and WT (n = 3) mice. Total RNA from renal tissue samples was isolated using TRIzol reagent (Promega, Madison, WI, USA). RNA sequencing was performed using an Illumina HiSeq2500 system (CapitalBio Technology Inc., Beijing, China). To obtain circRNAs, the raw sequencing data were analyzed using FastQC and fastp software to obtain clean reads. After being aligned with the reference genome sequence using Tophat2 software, circRNAs were identified using the find_circ and CIRCexplorer2 toolset. circRNA annotation was performed using BEDTools, a powerful tool for genome arithmetic [15]. After normalizing the circRNA expression levels between samples using the calculated number of reads per kilobase per million mapped reads, the differences in circRNA expression levels among different samples were compared using the limma software package [16]. Differentially expressed circRNAs between BKS-DB/Nju and WT mice were defined as |log2fold change|≥ 1 and *p* value < 0.05.

The raw sequencing data were submitted to the Sequence Read Archive (SRA) database (https://www.ncbi.nlm.nih.gov/sra).

### Prediction of microRNA response elements and miRNA targets

MicroRNA response elements (MREs) on the sequences of differentially expressed circRNAs were predicted using the miRanda database. miRNA targets were predicted using microT-CDS from DIANA TOOLS [17, 18].

### Gene Ontology and Kyoto Encyclopedia of Genes and Genomes pathway enrichment analysis

Gene Ontology (GO) analysis (http://geneontology.org/) using DAVID Bioinformatics Resources [19] was performed to predict the biological functions of the miRNA targets. GO functional analysis is divided into three parts: molecular function, biological processes, and cellular components. GO analysis was used to annotate gene function based on the GO database to obtain all functions of the gene parameters.

Kyoto Encyclopedia of Genes and Genomes (KEGG) pathway analysis (http://www.genome.jp/kegg/) using DAVID Bioinformatics Resources [19] was used to predict the pathways involved in miRNA targets.

### qRT-PCR

Total RNA from renal tissues was isolated using TRIzol. Reverse transcription was performed to obtain cDNA using M-MLV (Promega). qPCR was performed using SYBR GREEN qPCR Super Mix (Invitrogen, Carlsbad, CA, USA) on an ABI PRISM 7500 Sequence Detection System (Foster City, CA, USA). Divergent primers were designed to distinguish between circRNA transcripts and canonical linear transcripts according to the method reported by Panda et al. [20]. The primer sequences for all circRNAs verified by qRT-PCR are listed in Additional File 1. Agarose gel electrophoresis was performed to determine the length of the PCR product. The Sanger DNA sequence of the PCR amplification product was used for the validation of circRNA.

### Statistical analysis

All data are expressed as mean ± standard deviation (mean ± SD). Comparisons between data from the two groups were analyzed using the Student's *t*-test and SPSS software (version 19.0; IBM Corp., Armonk, NY, USA). Statistical significance was set at *p* < 0.05.

## Results

### BKS-DB/Nju mice are suitable as a model of diabetic nephropathy

**Fig. 1:**
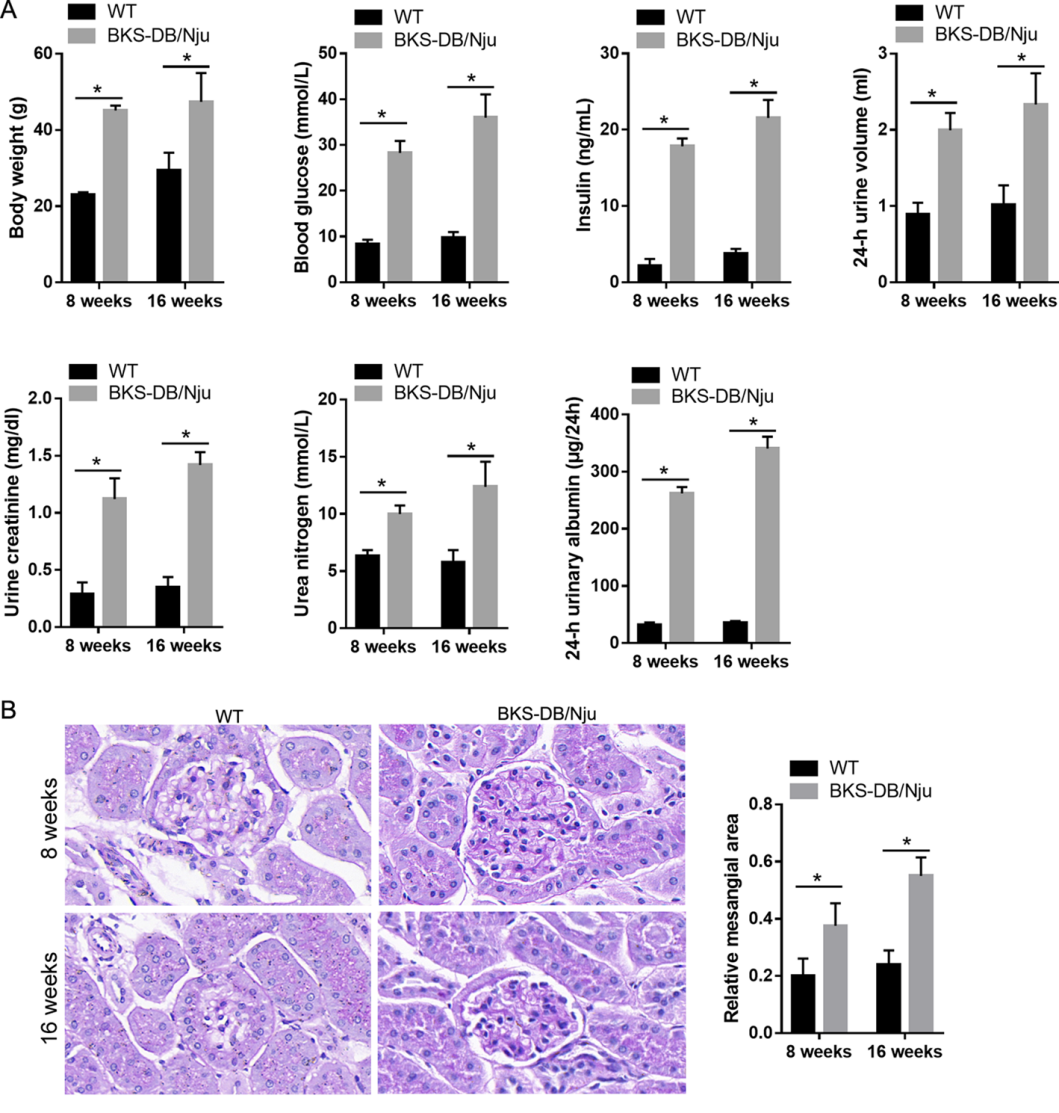
Comparisons between diabetic nephropathy mice BKS-DB/Nju and wild-type (WT) mice at both 8 weeks and 16 weeks of age. **A** Comparisons of body weights and blood and urine biochemical parameters. **B** Periodic acid-Schiff (PAS) staining results of renal tissues. Representative graphs of PAS staining are shown at the top of the figure. Statistical results of relative mesangial area are shown on the right of the figure. Mesangial expansion was prominent in the glomeruli of BKS-DB/Nju mice. *P < 0.05, n = 10

As shown in Fig. 1A, the body weights, blood glucose, insulin, urine creatinine, urea nitrogen, 24-h urinary albumin levels, and 24-h urine volume of diabetic BKS-DB/Nju mice were significantly higher than those of their nondiabetic WT littermates at both 8 weeks and 16 weeks of age. Additionally, the results of PAS staining revealed that the relative mesangial areas of BKS-DB/Nju mice were significantly higher than those of WT mice at both 8 weeks and 16 weeks of age, indicating that mesangial expansion was prominent in the glomerulus of BKS-DB/Nju mice (Fig. 1B). Together, these results indicate that BKS-DB/Nju mice are suitable for use as a model of diabetic nephropathy.

### Differentially expressed circRNAs

**Fig. 2:**
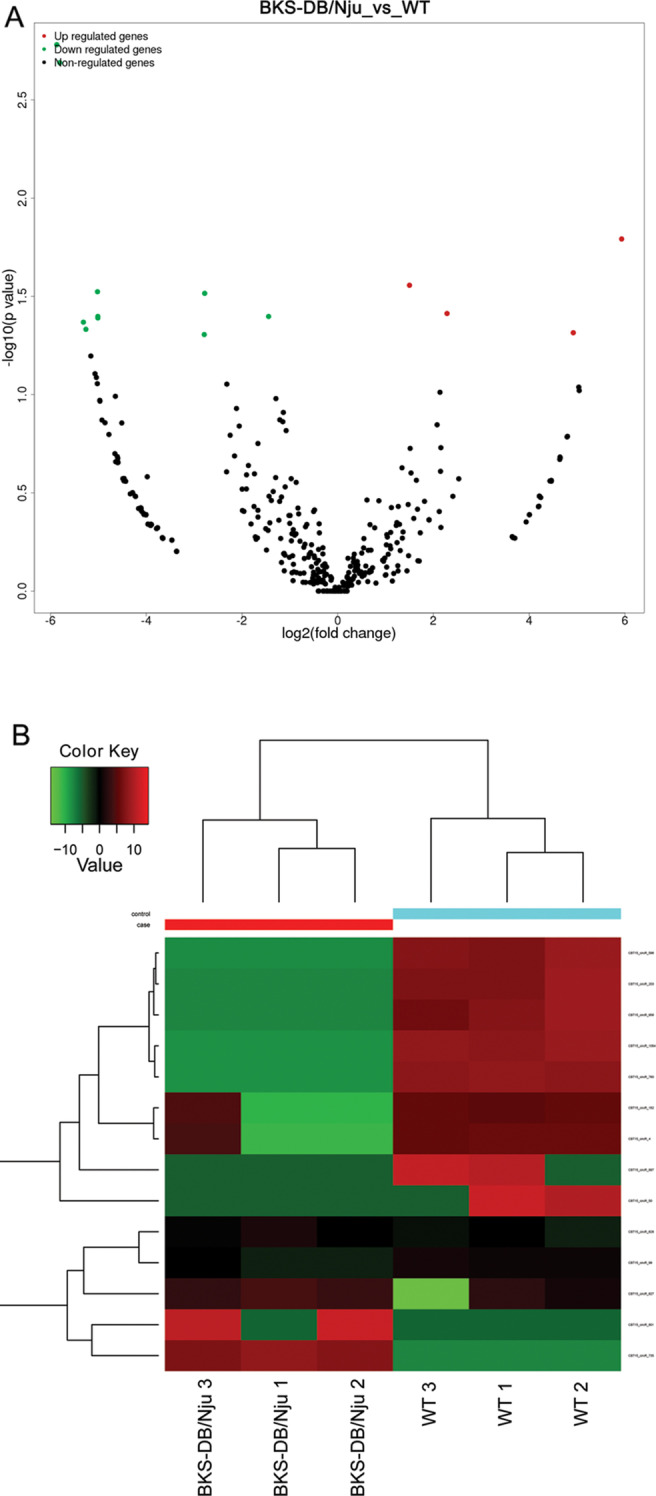
Differential expression analysis of circular RNAs (circRNAs). **A** Volcano plot. Red dots represent upregulated differential circRNAs, green dots represent downregulated differential circRNAs, and black dots represent circRNAs with no significant difference. **B** Hierarchical cluster analysis showing the 14 differentially expressed circRNAs

The raw sequencing data were deposited in the SRA database with the reference number PRJNA713921 (https://www.ncbi.nlm.nih.gov/sra/PRJNA713921). Among all identified circRNAs (see Additional File 2), four circRNAs were upregulated and 10 were downregulated in BKS-DB/Nju mouse kidneys compared to those in WT mouse kidneys (Fig. 2). Detailed information on the differentially expressed circRNAs is shown in Table 1. Notably, only circR_50, circR_182, and circR_627 have been previously reported in the circRNA dataset circBase. The IDs of circR_50, circR_182, and circR_627 in circBase are mmu_circ_0000130, mmu_circ_0000242, and mmu_circ_0000943, respectively.

**Table 1:** Detailed information of differentially expressed circular RNAs (circRNAs) identified by high-throughput RNA sequencing

circRNA number	Position	Fold change	Regulation	Gene Symbol	Exon information
circR_1084	chr6: 86,944,356–86,967,373	− 5.87	Down	Aak1	86,944,357–86,944,478;86,946,104–86,946,185; 86,946,861–86,946,993; 86,949,542–86,949,645; 86,950,781–86,950,860; 86,955,104–86,955,258; 86,956,259–86,956,572; 86,963,993–86,964,225; 86,965,477–86,965,634; 86,967,269–86,967,373
circR_760	chr2: 65,125,892–65,151,167	− 5.81	Down	Cobll1	65,125,893–65,126,117; 65,126,208–65,126,305; 65,133,579–65,133,807; 65,136,345–65,136,546; 65,150,979–65,151,167
circR_801	chr3: 146,463,184–146,493,843	5.93	Up	Spata1	146,463,185–146,463,255; 146,465,909–146,465,996; 146,468,106–146,468,204; 146,469,625–146,469,803; 146,475,256–146,475,381; 146,476,194–146,476,302; 146,481,128–146,481,209; 146,481,767–146,481,860; 146,487,200–146,487,419
ircR_628	chr19: 28,530,872–28,540,408	1.50	Up	Glis3	28,530,873–28,531,986; 28,540,201–28,540,408
circR_596	chr18: 74,577,454–74,580,534	− 5.02	Down	Myo5b	74,577,455–74,577,626; 74,580,390–74,580,534
circR_4	chr1: 119,567,795–119,579,077	− 2.78	Down	Epb41l5	146,463,185–146,463,255; 146,465,909–146,465,996; 146,468,106–146,468,204; 146,469,625–146,469,803; 146,475,256–146,475,381; 146,476,194–146,476,302
circR_627	chr19: 28,530,872–28,531,986	2.28	Up	Glis3	28,530,873–28,531,986
circR_956	chr5: 14,520,841–14,540,801	− 5.01	Down	Pclo	14,520,842–14,522,324; 14,539,410–14,540,801
circR_99	chr1: 66,801,048–66,802,168	− 1.44	Down	Kansl1l	66,801,049–66,802,168;
circR_203	chr11: 54,909,671–54,910,350	− 5.01	Down	Gpx3	54,909,672–54,910,350
circR_50	chr1: 189,786,635–189,798,681	− 5.31	Down	Ptpn14	189,786,636–189,786,948; 189,798,512–189,798,681
circR_897	chr4: 44,133,639–44,152,553	− 5.26	Down	Rnf38	44,133,640–44,133,761; 44,134,890–44,134,974; 44,137,555–44,137,661; 44,138,670–44,138,831; 44,142,295–44,142,465; 44,143,448–44,143,615 44,149,025–44,149,238; 44,152,360–44,152,553
circR_735	chr2: 25,952,677–25,966,948	4.92	Up	Camsap1	25,952,678–25,952,819; 25,956,247–25,956,327; 25,965,663–25,965,824; 25,966,686–25,966,948
circR_182	chr11: 20,725,684–20,727,639	− 2.79	Down	Aftph	20,725,685–20,727,639

### Verification of differentially expressed circRNAs during diabetic nephropathy

**Fig. 3:**
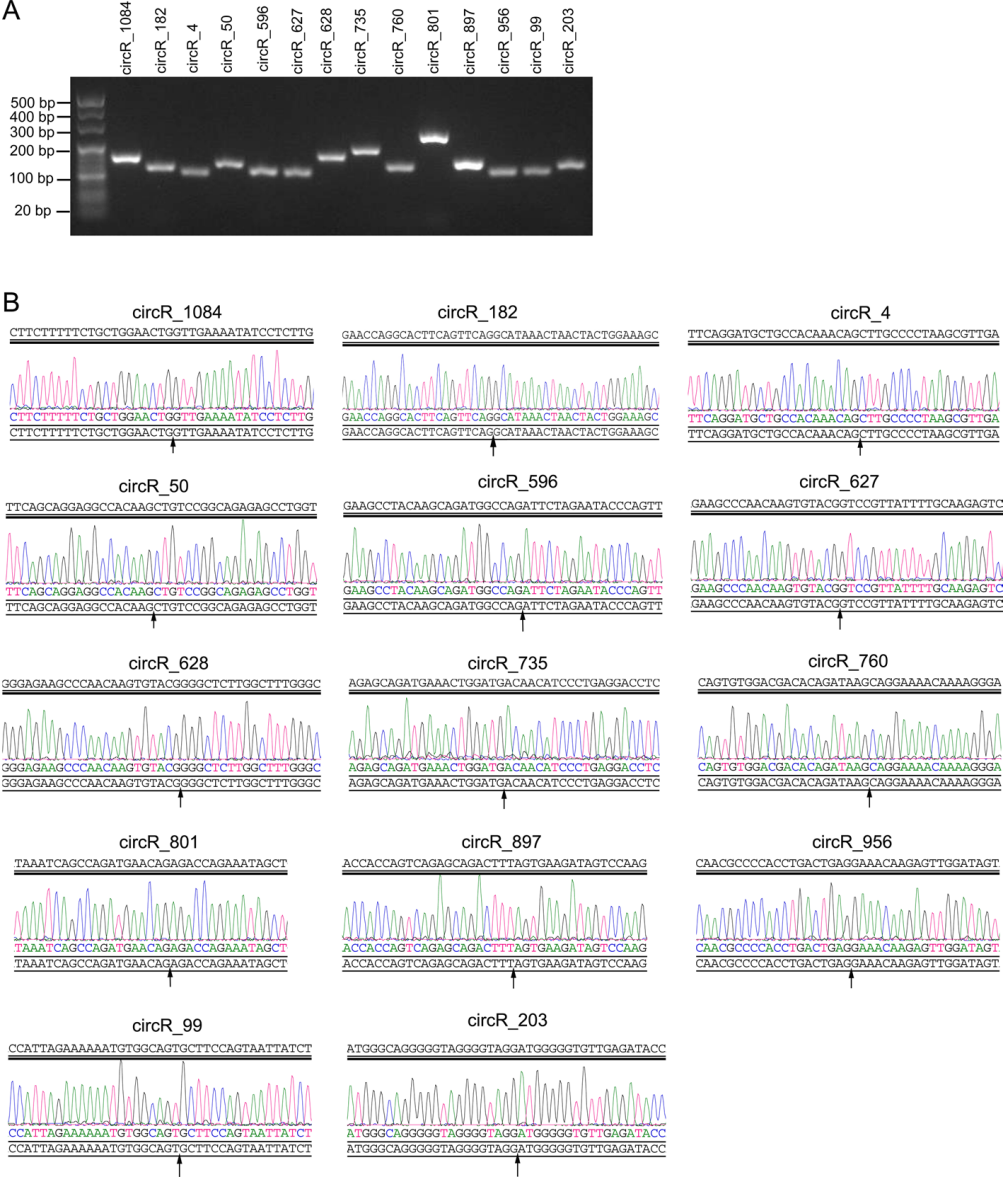
Results of agarose gel electrophoresis analysis and DNA sequence of polymerase chain reaction (PCR) amplification products. **A** Agarose gel electrophoresis analysis showed the length of PCR amplification products. Lane 1 is the DNA marker. **B** Sequence around the splice junction verified by DNA sequencing. Black arrows indicate the splice junction of circular RNAs (circRNA)

**Fig. 4:**
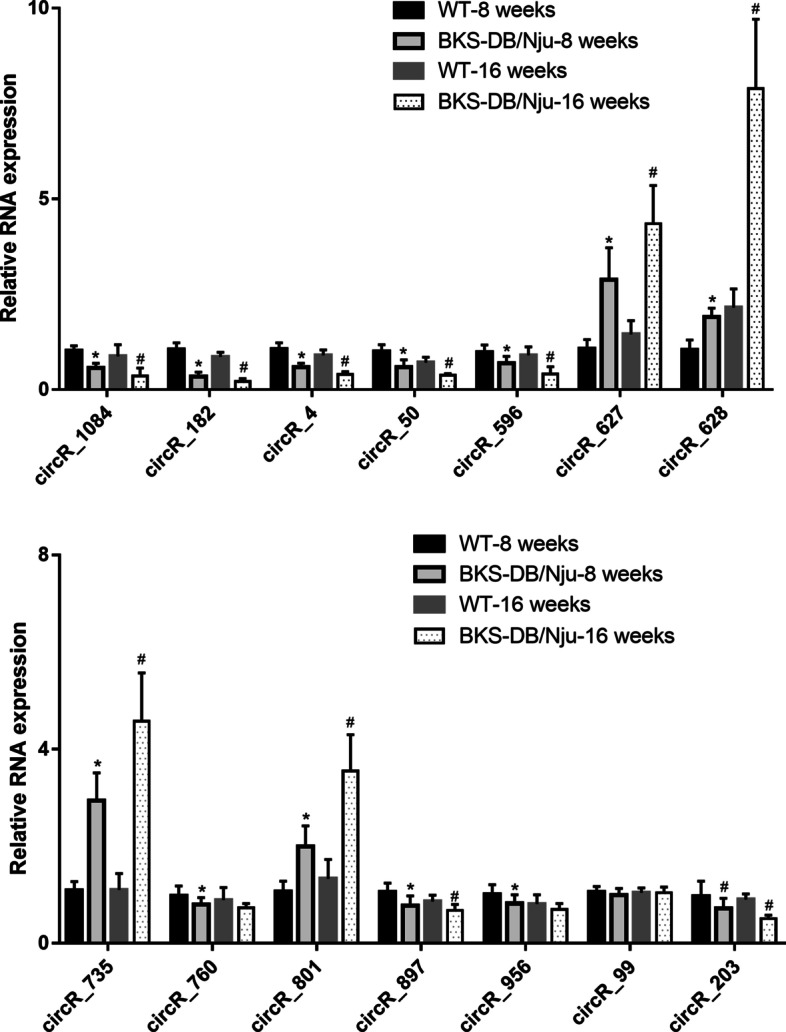
Expression levels of 14 circular RNAs (circRNAs) in the renal tissues obtained from both diabetic nephropathy mice BKS-DB/Nju and wild-type (WT) mice (8 weeks old and 16 weeks old). *P < 0.05, BKS-DB/Nju-8 weeks vs. WT-8 weeks; ^#^*P* < 0.05, BKS-DB/Nju-16 weeks vs. WT-16 weeks; n = 10

The expression of 14 differentially expressed circRNAs was verified by qRT-PCR. The length of the PCR product was checked by agarose gel electrophoresis (Fig. 3A), and the length was the same as expected in Additional File 1. The PCR product was sequenced, and the sequence around the splice junction is shown in Fig. 3B. The qRT-PCR results revealed that the expression levels of circR_1084, circR_182, circR_4, circR_50, circR_596, circR_897, and circR_203 were downregulated in the renal tissues of 8- and 16-week-old BKS-DB/Nju mice compared to those in WT mice (Fig. 4). The expression levels of circR_760 and circR_956 were downregulated only in the renal tissues of 8-week-old BKS-DB/Nju mice compared to those in WT mice (Fig. 4). The expression levels of circR_627, circR_628, circR_735, and circR_801 were upregulated in the renal tissues of 8- and 16-week-old BKS-DB/Nju mice compared to those in WT mice (Fig. 4). The circR_99 expression level showed no obvious changes (Fig. 4).

### MREs of differentially expressed circRNAs

Some circRNAs contain MREs. CircRNAs can bind to miRNAs through MREs and act as competitive endogenous RNAs (ceRNAs), thereby relieving the effects of miRNAs on their target genes. To evaluate the potential functions of differentially expressed circRNAs as ceRNAs, we predicted the MREs of the differentially expressed circRNAs listed in Table 1. All results are shown in Additional File 3. Moreover, we predicted the miRNA target genes of all miRNAs shown in Additional File 3, and the results are shown in Additional File 4.

### Predicted biological functions of miRNA targets

To predict the biological functions of differentially expressed circRNAs, GO and KEGG pathway analyses for the miRNA targets shown in Additional File 4 were performed. The top 100 terms for GO-enriched biological processes, cellular components, and molecular functions are shown in Additional File 5. The details of all GO-enriched items are shown in Additional File 6. All terms for KEGG pathway analysis for miRNA targets are shown in Additional File 7, and the details of the KEGG pathway analysis are shown in Additional File 8.

## Discussion

Diabetic nephropathy is one of the most important complications observed in patients with diabetes [4, 21]. As the etiology and pathogenesis of diabetic nephropathy are unclear, studies examining the pathogenesis of diabetic nephropathy are ongoing. Several studies have indicated that circRNAs play crucial regulatory roles in many human diseases and in normal physiology [9–11]. circRNA-HIPK3 [12] and circRNA-cPWWP2A [13] have been reported to regulate diabetic nephropathy, indicating that circRNAs may be involved in diabetic nephropathy development, and could be used as new therapeutic targets for diabetic nephropathy. To date, no study has focused on the comprehensive expression profile of circRNAs in diabetic mouse kidneys. Therefore, we aimed to identify differentially expressed circRNAs in diabetic mouse kidneys to explore the potential roles of dysregulated circRNAs in the development of diabetic nephropathy.

In this study, BKS-DB/Nju mice were used as a diabetic nephropathy mouse model. BKS-DB/Nju mice are transgenic and constructed through the knockout of exon 19 of the leptin receptor (Lepr) using CRISPR/Cas9 technology on a C57BL/KsJ background. Lepr, also known as a diabetes gene (db), is closely associated with obesity, hypertension, diabetes, and lipid metabolism disorders [22–24]. Compared to previously developed type 2 diabetic db/db mouse models [25, 26], BKS-DB/Nju represents a new diabetic mouse model that has not been widely used. We found that the body weights and blood and urine biochemical parameters of BKS-DB/Nju mice are characteristic of diabetic mice. Hence, we hypothesized that BKS-DB/Nju mice would provide a suitable diabetic nephropathy mouse model. However, as BKS-DB/Nju mice have not been extensively used, this is a limitation of the present study, and the results should be verified in a type 2 diabetic db/db mouse model.

Among all identified circRNAs, four circRNAs were upregulated and 10 were downregulated in diabetic mouse kidneys compared to the levels in nondiabetic mouse kidneys. The differential expression profile in the renal tissues of BKS-DB/Nju mice indicates that these circRNAs may regulate diabetic nephropathy. Further studies should be conducted to confirm the expression levels and specific functions of these differentially expressed circRNAs. Nevertheless, our results provide a novel theoretical basis for further studies examining circRNA functions in the context of diabetic nephropathy regulation.

To further verify the differential expression of circRNAs, qRT-PCR was performed to detect 14 differentially expressed circRNAs. The results of qRT-PCR showed that expression level of only circR_99 had no significant change in the renal tissues of 8-week-old BKS-DB/Nju mice compared to those in WT mice. The high degree of consistency between RNA sequencing and qRT-PCR demonstrated the reliability and veracity of our results. In addition, we found that the expression of most differentially expressed circRNAs at 8 weeks old was also dysregulated at 16 weeks old. Eight-week-old BKS-DB/Nju mice were at an early stage of diabetic nephropathy. The consistently high or low expression of these circRNAs with increasing week age of diabetic mice indicated that these circRNAs may be closely related to the progression of diabetic nephropathy. In future studies, we will verify these differentially expressed circRNAs using other diabetic nephropathy models, such as type 2 diabetic db/db mice. Moreover, functional experiments should be performed in vitro and in vivo to further evaluate the potential role of differentially expressed circRNAs in the etiology and pathogenesis of diabetic nephropathy.

An increasing number of studies have suggested that many circRNAs contain MREs, which play a role through the circRNA-miRNA-mRNA regulatory network [27, 28]. In this regulatory network, circRNAs function as ceRNAs to regulate the gene expression of miRNA targets. Therefore, it is rational to predict the biological functions of circRNAs based on miRNA target genes. In the present study, we predicted all MREs of differentially expressed circRNAs and predicted the biological functions of miRNA target genes using GO and KEGG pathway analyses. Numerous GO and KEGG pathways were identified. Among all these pathways, some enriched GO items have been reported to be involved in diabetic nephropathy, such as cAMP-mediated signaling [29], p53 binding [30], and insulin receptor binding [31]. Moreover, some enriched pathway items have been reported to be involved in diabetic nephropathy, such as the MAPK, mTOR, and Jak-STAT signaling pathways [32]. Therefore, the results of GO and KEGG pathway analyses suggest that differentially expressed circRNAs may be involved in diabetic nephropathy. However, the identification of numerous circRNA-miRNA-mRNA regulatory networks indicated that additional studies are required to elucidate the circRNA-mediated mechanisms underlying the pathogenesis of diabetic nephropathy. Regardless, predictions based on the biological functions of miRNA target genes can help us understand the functions of circRNAs.

## Conclusions

This is the first study to report the use of RNA sequencing to identify the comprehensive circRNA expression profile in the kidneys of diabetic BKS-DB/Nju mice. After verification by qRT-PCR, we found that circR_1084, circR_182, circR_4, circR_50, circR_596, circR_897, and circR_203 were downregulated, whereas circR_627, circR_628, circR_735, and circR_801 were upregulated in the renal tissues of BKS-DB/Nju mice. These differentially expressed circRNAs may serve as candidate biomarkers for diabetic nephropathy. Collectively, the results of this study provide a novel theoretical basis for further investigations on the regulatory roles of circRNAs in the etiology and pathogenesis of diabetic nephropathy.

## Supplementary Information

**Additional file 1:** Primer sequences of all circular RNAs (circRNAs) verified by quantitative reverse transcriptase polymerase chain reaction (qRT-PCR)

**Additional file 2:** All identified circular RNAs (circRNAs) in three diabetic BKS-Leprem2Cd479Nju (BKS-DBNju) mice and three nondiabetic littermates of C57BLKsJ wild-type (WT) mice

**Additional file 3:** MicroRNA response elements of all differentially expressed circular RNAs (circRNAs)

**Additional file 4:** MicroRNA (miRNA) target genes were predicted using microT-CDS from DIANA TOOLS

**Additional file 5:** Bar plot with gradient of Gene Ontology TOP 100

**Additional file 6:** Gene Ontology results obtained using DAVID Bioinformatics Resources

**Additional file 7:** Dot plot of all enriched KEGG pathways

**Additional file 8:** KEGG pathway annotation results obtained using DAVID Bioinformatics Resources

## Data Availability

The datasets used and/or analyzed during the current study are available from the corresponding author upon reasonable request.

## Abbreviations

CircRNAs: Circular RNAs
BKS-DB/Nju: BKS-Lepr^em2Cd479^/Nju
WT: Wild-type littermate C57BL/KsJ
PAS: Periodic acid-Schiff
GO: Gene ontology
KEGG: Kyoto Encyclopedia of Genes and Genomes
qRT-PCR: Quantitative reverse transcriptase polymerase chain reaction
db: Diabetes gene
Lepr: Leptin receptor
