# Supplementary material for: Circular RNA expression profile in transgenic diabetic mouse kidneys
Source: Cell Mol Biol Lett. 2021 Jun 7;26:25. doi: 10.1186/s11658-021-00270-z (PMC8182942; doi:10.1186/s11658-021-00270-z)
Supplement: Supplementary file 1 — Additional file 1: Primer sequences of all circular RNAs (circRNAs) verified by quantitative reverse transcriptase polymerase chain reaction (qRT-PCR) [file 11658_2021_270_MOESM1_ESM.docx]

| circRNA  number | forward primer (5’-3’) | reverse primer (5’-3’) | product length |
| --- | --- | --- | --- |
| circR_1084 | AGGCTTCCAGCCAACACAAG | CATTGACTCCCTCGGCCTGT | 159 bp |
| circR_50 | TGCCAAATGTGTCACGGCTT | GCACCCCTGAGATGGCCTTA | 134 bp |
| circR_596 | TGACATGGACCCCCACATCT | ATTCCGCAAGAATGGCACCT | 111 bp |
| circR_627 | GGACTGGCTGCCCTAGAAGA | TCGTGGACACCAGAGACTCT | 106 bp |
| circR_628 | AAGCCTTTCAACGCACGGTA | CATCATGGGACTGCTGGTGAC | 158 bp |
| circR_735 | GATGGCGCTGCTCTCTTAGC | GCTGCAGACACGGCAATACA | 180 bp |
| circR_760 | CAAGAAGAGACGTGCCCCAC | AGAGACATCAGTGCCCTTCGT | 121 bp |
| circR_801 | CACCTTTCCGACCTCCGATG | GCGACATGTTGTCCAGTGTCT | 235 bp |
| circR_897 | GCCCTGTTAAACTTGGCGGA | GGCGCTGTCTCTTAGGACTT | 137 bp |
| circR_956 | ACCCAACGCCCCACCTGACT | GTCCCCCTCAGCATGGTCTT | 105 bp |
| circR_99 | GGACTCTGACGCCACCGATA | GGCGAATCCTGGAGATAATT | 101 bp |
| circR_203 | TGGGGGTAGAATGACTGGGAA | TCTCACTCTGTCCACTGGTA | 120 bp |

Additional file 1 Primer sequences of all circular RNAs (circRNAs) verified by quantitative reverse transcriptase polymerase chain reaction (qRT-PCR)
