# Supplementary figures and images for: Circular RNA expression profile in transgenic diabetic mouse kidneys
Source: Cell Mol Biol Lett. 2021 Jun 7;26:25. doi: 10.1186/s11658-021-00270-z (PMC8182942; doi:10.1186/s11658-021-00270-z)

GO BarPlot

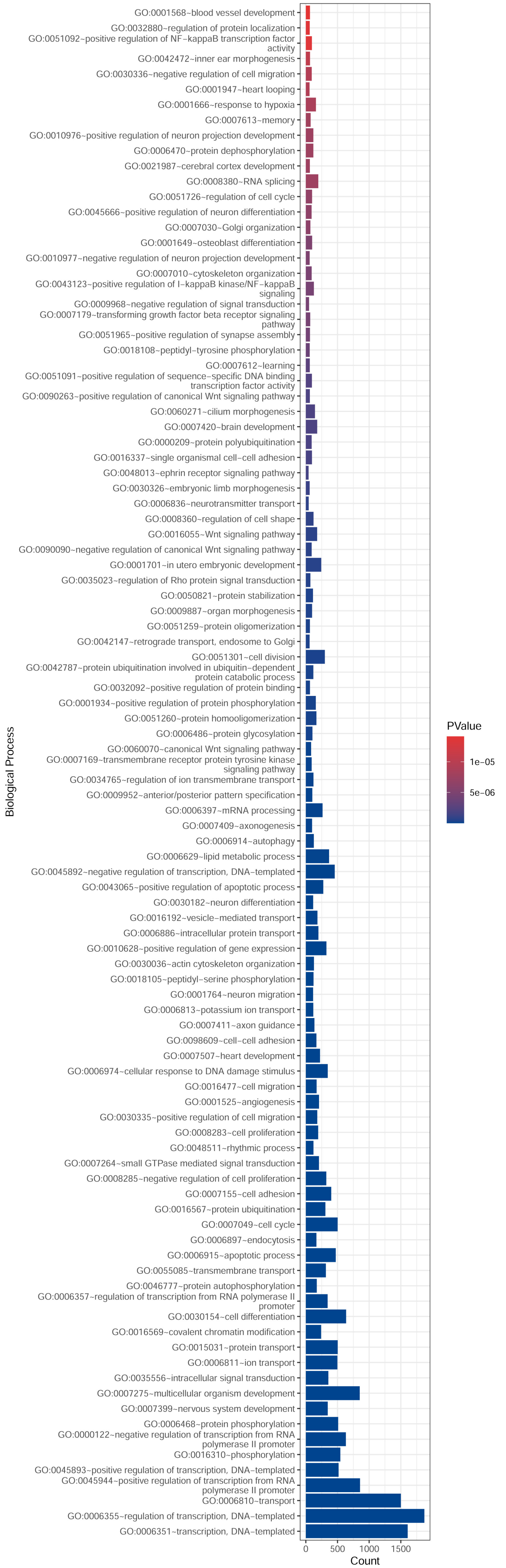

GO BarPlot

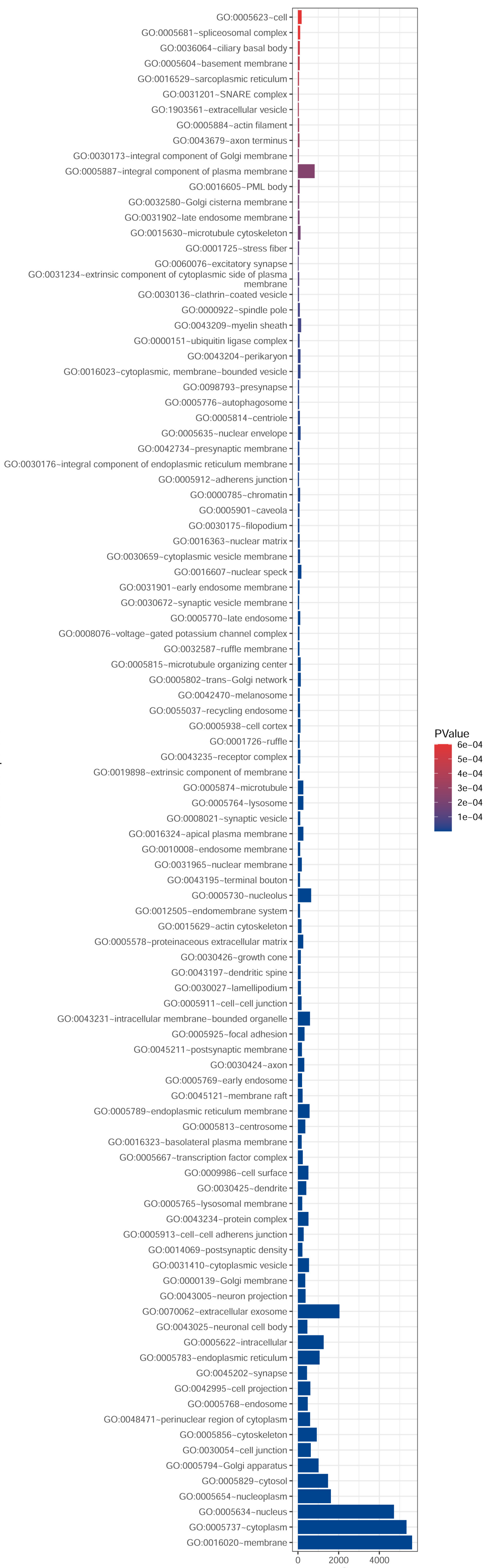

GO BarPlot

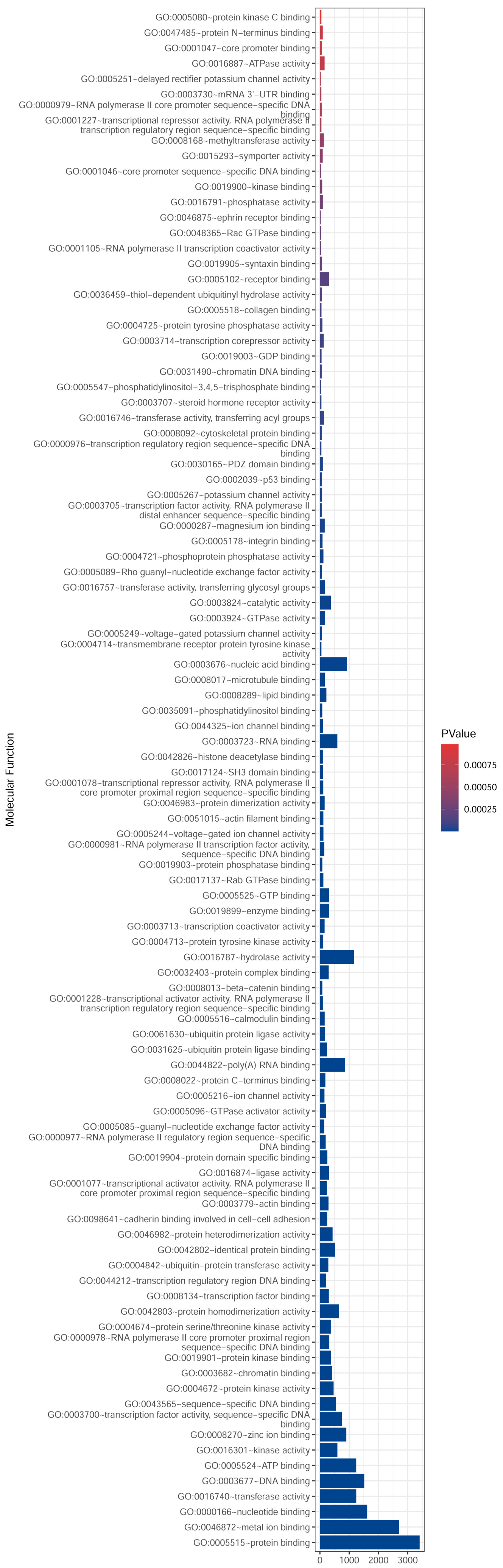

Supplement: Supplementary file 5 — Additional file 5: Bar plot with gradient of Gene Ontology TOP 100 [file 11658_2021_270_MOESM5_ESM.pdf]

Enriched Pathways

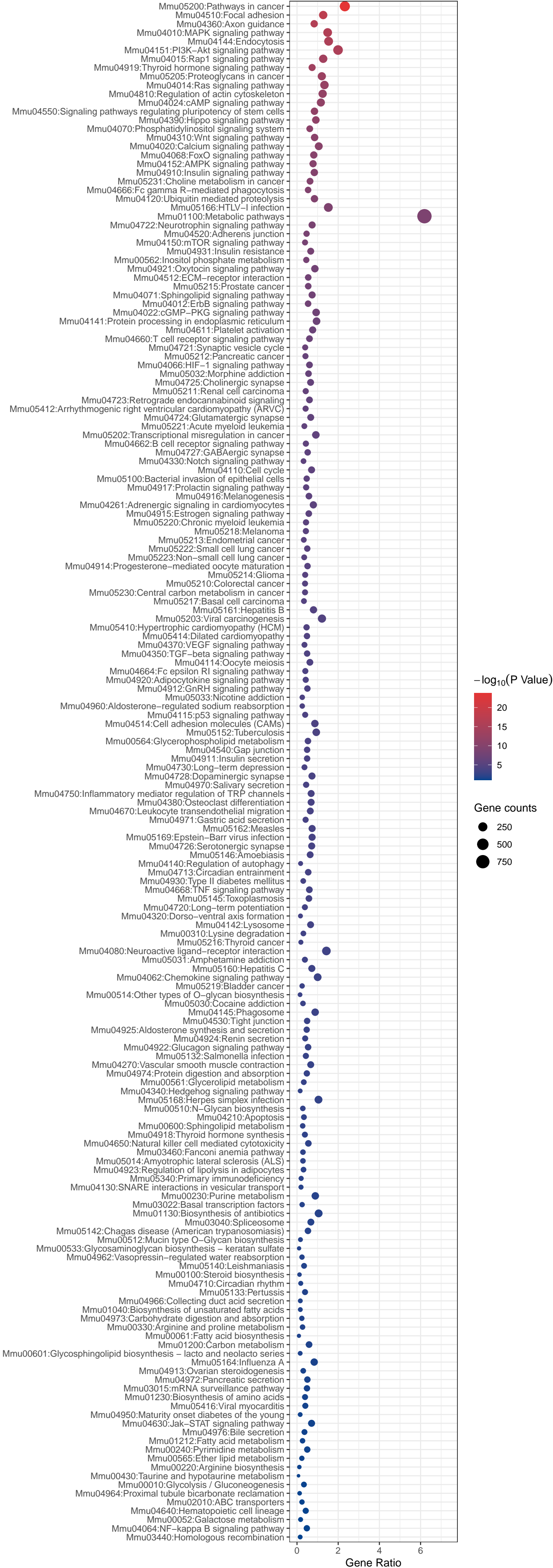

Supplement: Supplementary file 7 — Additional file 7: Dot plot of all enriched KEGG pathways [file 11658_2021_270_MOESM7_ESM.pdf]
